# Durability of improvement in post-traumatic stress disorder symptoms and absence of harmful effects or drug dependency after 3,4-methylenedioxymethamphetamine-assisted psychotherapy: a prospective long-term follow-up study

**DOI:** 10.1177/0269881112456611

**Published:** 2013-01

**Authors:** Michael C Mithoefer, Mark T Wagner, Ann T Mithoefer, Lisa Jerome, Scott F Martin, Berra Yazar-Klosinski, Yvonne Michel, Timothy D Brewerton, Rick Doblin

**Affiliations:** 1 Private Practice, Mount Pleasant, SC, USA; 2 Clinical Research for Multidisciplinary Association for Psychedelic studies (MAPS), Mount Pleasant, SC, USA; 3 Department of Neuroscience, Medical University of South Carolina, Charleston, SC, USA; 4 Multidisciplinary Association for Psychedelic Studies, Santa Cruz, CA, USA; 5 Private Practice, Cleveland, OH, USA; 6 Multidisciplinary Association for Psychedelic Studies, Santa Cruz, CA, USA; 7 Private Consultant in Biostatistics, Daniel Island, SC, USA; 8 Medical University of South Carolina, Mount Pleasant, SC, USA; 9 Multidisciplinary Association for Psychedelic Studies, Belmont, MA, USA

**Keywords:** MDMA, post-traumatic stress disorder, PTSD, psychedelic drugs, ecstasy, mental health, victimization, long-term outcome, treatment resistance, psychotherapy methods, pharmacotherapy

## Abstract

We report follow-up data evaluating the long-term outcomes for the first completed trial of 3,4-methylenedioxymethamphetamine (MDMA)-assisted psychotherapy for chronic, treatment-resistant post-traumatic stress disorder (PTSD) (Mithoefer et al., 2011). All of the 19 subjects who received MDMA-assisted treatment in the original trial participated in the long-term follow-up (LTFU), with 16 out of 19 completing all of the long-term outcome measures, which were administered from 17 to 74 months after the original study’s final MDMA session (mean = 45.4; SD = 17.3). Our primary outcome measure used was the Clinician-Administered PTSD Scale (CAPS). Secondary outcome measures were the Impact of Events Scale-Revised (IES-R) and the Neuroticism Extroversion Oppenness Personality Inventory-Revised (NEO PI-R) Personality Inventory. We also collected a long-term follow-up questionnaire. Results for the 16 CAPS completers showed there were no statistical differences between mean CAPS score at LTFU (mean = 23.7; SD = 22.8) (*t*_matched_ = 0.1; df = *15*, *p* = 0.91) and the mean CAPS score previously obtained at Study Exit (mean = 24.6, SD = 18.6). On average, subjects maintained statistically and clinically-significant gains in symptom relief, although two of these subjects did relapse. It was promising that we found the majority of these subjects with previously severe PTSD who were unresponsive to existing treatments had symptomatic relief provided by MDMA-assisted psychotherapy that persisted over time, with no subjects reporting harm from participation in the study.

## Introduction

Post-traumatic Stress Disorder (PTSD) can be a chronic, severely disabling condition causing sustained loss of functionality, accompanied by high rates of medical and psychiatric co-morbidity and risk of suicide (Perkonigg et al., 2000; Breslau, 2001; Kessler et al., 2005; Seal et al., 2010). Existing pharmacological and psychotherapeutic treatments for PTSD are effective for many, but not all sufferers (Brady et al., 2000a; Marshall et al., 2001; Ursano et al., 2004; Foa et al., 2009). Due to the rate of treatment resistance, the need for research into a wider array of more effective treatments is widely recognized (Ursano et al., 2004; Foa et al., 2009; Stein et al., 2009).

Prior to its placement into the most restrictive category of drug regulation in the US and internationally, uncontrolled published reports suggested that the substituted phenethylamine 3,4-methylenedioxymethamphetamine (MDMA), when administered in conjunction with psychotherapy, could yield substantial benefits for those afflicted with a variety of disorders (Greer and Tolbert, 1986). When we published the first randomized controlled trial of MDMA-assisted psychotherapy, our results demonstrated that there were positive effects on PTSD symptom severity by the end of the treatment program (Mithoefer et al., 2011).

MDMA is hypothesized to support and enhance psychotherapy by increasing the subject’s access to emotionally-upsetting material, modulating the associated level of arousal and strengthening the therapeutic alliance. MDMA produces unique changes in emotions in humans (Cami et al., 2000; Liechti et al., 2001; Harris et al., 2002; Tancer and Johanson, 2003; Bedi et al., 2010; Studerus et al., 2010; Kirkpatrick et al., 2012) through a complex combination of pharmacological effects. MDMA is not only a monoamine releaser with particularly prominent effects on serotonin (Liechti et al., 2000; Liechti and Vollenweider, 2001; Setola et al., 2003; Farre et al., 2007; Kolbrich et al., 2008), but it also elevates serum oxytocin (Wolff et al., 2006; Dumont et al., 2009), which is a neuropeptide believed to play a role in affiliation and bonding in mammals (Pitman et al., 1993; Bartz and Hollander, 2006; Olff et al., 2010). Brain imaging studies show there is reduced amygdalar activity after MDMA administration (Gamma et al., 2000), plus changes in the response to angry and happy facial expressions (Bedi et al., 2009). Healthy volunteers given MDMA were better able to spot positive facial expressions and found it harder to spot negative ones, when compared with placebo (Hysek et al., 2012). Taken together, these findings suggest that MDMA may enhance the therapeutic alliance by increasing the likelihood of detecting positive expressions and finding them rewarding, while at the same time reducing the chance of excessive reactivity to fleeting or unintended expressions of anger or disapproval (Rauch et al., 2006). These effects may combine to increase the effectiveness of psychotherapy for PTSD, by increasing self-acceptance, promoting interpersonal trust with therapists and catalyzing the effective processing of emotionally-distressing material. Recent investigations also support the potential for MDMA as a treatment for people with PTSD (Bouso et al., 2008; Mithoefer et al., 2011).

Evaluation of longer-term outcomes, though infrequently reported in the psychiatric literature, may contribute significantly to the understanding and treatment of chronic mental illnesses like PTSD and their associated morbidity and disability. For example, long-term follow-up (LTFU) studies could help to formulate treatment guidelines, permit evaluation of the rates of sustained symptom reduction or remission, predict the need for maintenance treatment, permit the assessment of long-term tolerability and help rule out a placebo response (Price et al., 2008).

A number of studies have tracked the course of PTSD over time (Peleg and Shalev, 2006), but the evaluations of treatment effects in clinical trials typically has ended within several months after treatment. Only a minority of psychiatric treatment trials have followed people from one-half year up to four years after their initial treatment, using open-label designs to assess the patients after they completed randomized or blinded studies. So far, pharmacotherapy with sertraline (Davidson et al., 2001; Rapaport et al., 2002) and nefazodone (Hertzberg et al., 2002) show sustained efficacy at LTFU, at one-half and 3–4 years, respectively, given continuous drug treatment over these intervals. An investigation of the treatment effects following a 4-month intensive inpatient program of psychotherapy and supportive treatment for PTSD patients revealed that the reduction in symptoms of PTSD had not persisted at 18 months (Johnson et al., 1996). LTFU studies of psychotherapies such as Eye Movement Desensitization and Reprocessing (EMDR) (Edmond and Rubin, 2004; Van der Kolk et al., 2007; Zimmermann et al., 2007; Hogberg et al., 2008), as well as cognitive behavioral and psycho-educational treatments (Solomon et al., 2005; Dorrepaal et al., 2010) show that benefits can be maintained. Practice guidelines for the treatment of PTSD accept the need for replication of previous studies, as well as the need for novel treatments, more specifically pharmacological agents that could augment psychotherapy (Benedek et al., 2009).

In order to address the above issues, we added LTFU of the 19 study subjects who received MDMA-assisted psychotherapy, as an amendment to the initial study design. Here we report the results of our extended surveillance of these subjects.

## Methods and materials

### Subjects

In the initial study (Mithoefer et al., 2011), 20 subjects with treatment-resistant PTSD (in most cases from sexual abuse or assault) were randomly assigned to psychotherapy with the active drug (*n* = 12) or with an inactive placebo (psychotherapy-only; *n* = 8), each administered during two 8-hour sessions scheduled 3–5 weeks apart, accompanied by weekly non-drug sessions. The MDMA-assisted sessions were conducted in a comfor setting, in which participants were encouraged to spend considerable time focused inward without talking, alternated with time spent talking to the therapists. The therapists took a non-directive approach to supporting their subjects in processing trauma-related material. More information concerning the nature of the psychotherapy is found in the Mithoefer et al. (2011) study, as well as in the author’s treatment manual (MAPS, 2011).

At the end of this controlled study, the participants who had received the psychotherapy-only treatment were offered open-label MDMA-assisted psychotherapy, using the same schedule of sessions as were used in the controlled study protocol. Of the eight therapy-only subjects, seven accepted and completed the crossover arm of the study, which resulted in 19 of the 20 study subjects receiving the MDMA-assisted psychotherapy treatment. The one therapy-only subject had recovered from PTSD symptoms with psychotherapy alone, and did not participate in the crossover. As allowed by a protocol amendment, the last eight subjects recruited (five in the double-blind stage and three in the crossover stage) also received a third MDMA-assisted psychotherapy session. This protocol change was sought because of tentative clinical impressions by the investigators that a third session would likely enhance the processing of trauma and the integration process that were essential to the treatment.

Subjects were mailed an informed consent form and a letter requesting their participation in the LTFU study. All 20 subjects from the initial study were recruited for LTFU and participated in the data collected herein. Detailed demographic information on the participants, including types of trauma, was published in the original report (Mithoefer et al., 2011).

**Figure 1.**
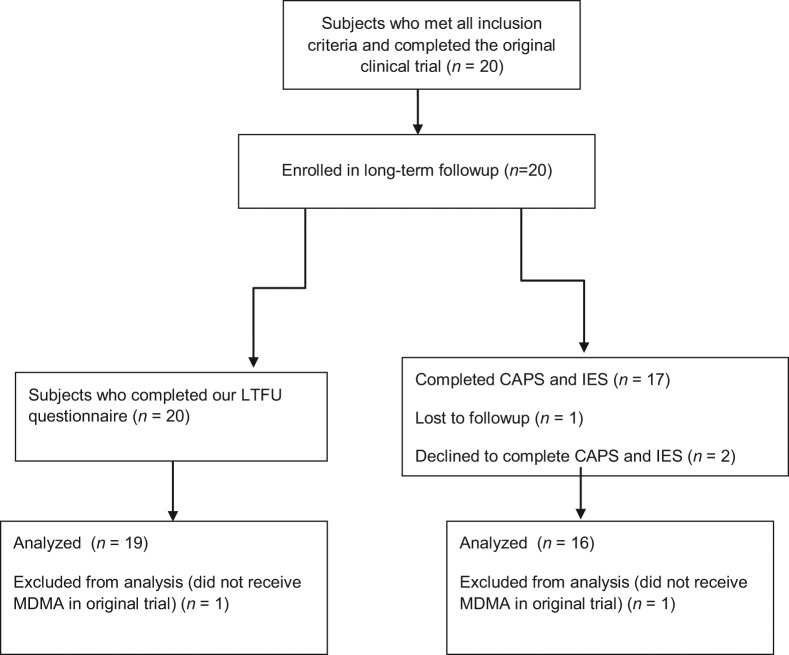
Study design with number of participants.

All 20 subjects completed our LTFU questionnaire, and 17 (14 women/3 men, mean age at enrollment = 42.3 yrs) completed the Clinician-Administered PTSD Scale (CAPS) and Impact of Events Scale-Revised (IES-R) as well (Figure 1). Because the purpose of our LTFU was to see if benefits from MDMA-assisted psychotherapy persisted over time, the data from the one subject who never received MDMA was excluded from the data analysis, but is reported below. Results focused on the 19 subjects (16 women/3 men, mean age at enrollment = 41.01) who received MDMA-assisted treatment, 16 of whom had also completed CAPS and IES-R measures.

There were three subjects who did not complete the measures: one moved and so was lost to follow-up after answering the LTFU questionnaire, but before completing the IES-R or scheduling administration of the CAPS; while the other two declined to repeat these two measures. Of the latter, one subject, who had previously shown the smallest reduction in CAPS score at the 2-month follow-up, was concerned that testing might trigger more symptoms and the other subject, who had responded well during the original study, declined to complete the measures again, citing stressful family matters as the reason for not devoting time nor attention to our current study.

Our study’s LTFU ranged from 17–74 months (mean = 45.4; SD = 17.3; *n* = 17) after the final MDMA session for the CAPS and IES-R administration, and 10–74 months (mean = 40.8; SD = 19.2; *n* = 20) after completion for the questionnaire. The original protocol and all of the protocol amendments were approved by an independent IRB (Copernicus Group IRB, Research Triangle Park, NC).

### Outcome measures

The CAPS, as in the original trial, remained the primary outcome measure. The CAPS yields a global symptom severity score, as well as a categorical ranking as to whether or not a subject meets DSM-IV-R criteria (American Psychiatric Association, 2000) for PTSD diagnosis (Weathers et al., 2001). The IES-R (Weiss and Marmar, 1996) is a global measure of psychological response to stress that we used as a secondary outcome measure.

We created the LTFU questionnaire for use in LTFU evaluations of MDMA-assisted psychotherapy. This questionnaire is designed to specifically capture the perceived benefit or harm of MDMA-assisted psychotherapy and changes in any areas not addressed by standard outcome measures, such as changes in relationships or creativity. The questionnaire measured the degree (with an ordinal scale of 1-Slight to 5-Large) and persistence (scale of 1-Small to 5-All) of the perceived benefits and/or harms of MDMA-assisted psychotherapy for PTSD. Additionally, the questionnaire included items addressing participant beliefs concerning the potential benefit of receiving an additional MDMA-assisted psychotherapy session, any psychiatric treatment after the study (whether psychotherapy or psychiatric medications), and their use of “ecstasy” (material represented as containing MDMA) and/or any other illicit psychoactive substances after study participation, plus any perceived changes in cognition after study participation. The participants were invited to write comments relating to their participation in the study. The full questionnaire is available online (MAPS, 2009) as supplemental material.

In conjunction with the questionnaire, subjects were informed in writing that the investigators had obtained a certificate of confidentiality, a document issued by the US Department of Health and Human Services in line with the National Institutes of Health Service Act, that protects research subjects from the legal consequences of providing investigators with sensitive information, such as information on past illegal drug use during the course of research (Health and Human Services, 2011). The participants were not specifically instructed nor requested to abstain from the use of ecstasy or any other drugs after completing the original study.

### Study design

In order to test the hypothesis that PTSD symptom improvement was sustained at LTFU, the post-treatment outcome scores at the 2-month follow-up were compared to LTFU outcome scores obtained in the present study, using independent *t* tests for the continuous global scores, independent Mann-Whitney *U* tests for the ordinal outcomes, and descriptive statistics for the questionnaire-based measurements.

Study subjects were mailed the LTFU questionnaire and an IES-R, to complete and mail back. Also, they were contacted to schedule administration of the CAPS in person or via phone, administered by the same independent rater who had administered the CAPS to all subjects in the original study. Our participants also completed the NEO-PI-R personality inventory (Costa and Macrae, 1992), but this NEO data will be presented in a separate publication, currently in preparation.

Because of our protocol amendment permitting an additional MDMA-assisted session, 8 subjects received three MDMA sessions and 11 subjects received two MDMA sessions prior to LTFU participation. Analysis of this small, internal pilot comparing the effects of two versus three MDMA-assisted sessions demonstrated that there was no statistical evidence of a difference in the participant’s LTFU outcomes (CAPS: *t*_independent_ = 0.2; df =14, *p* =0.83 and IES-R: *t*_independent_ = 0.7; df = 14, *p*= 0.48). Based on this analysis, LTFU scores were compared to the CAPS and IES-R scores obtained two months from the second MDMA-assisted session (2-month scores). These 2-month scores were the final short-term outcome time-point reported in our initial study (Mithoefer, 2011).

## Results

### CAPS and IES-R

We found that the mean CAPS and IES-R scores at LTFU for the 16 study completers were not statistically different from their 2-month (short-term) mean scores (Table 1).

**Table 1.** Early final study CAPS and IES-R Scores, 2 months after two MDMA-assisted sessions, versus the LTFU scores obtained in this study, for the same subjects.

CAPS	*n*	Mean	SD	*t*	df	*p*
2-month	16	24.6	18.6	0.1	15	.91
LTFU	16	23.7	22.8			

IES-R	*n*	Mean	SD	*t*	df	*p*

2-month	16	19.8	19.5	0.4	15	.72
LTFU	16	22.1	21.8			

CAPS: clinician-administered PTSD scale; IES-R: impact of events scale-revised; LTFU: long-term followup; MDMA: 3,4-methylenedioxymethamphetamine; PTSD: post-traumatic stress syndrome.

At LTFU, two subjects had CAPS scores above 50 (13%), which indicates relapse with moderate-to-severe PTSD symptoms, which is above the cut-off for original study entry. Using an intent-to-treat analysis, the three subjects who did not complete the CAPS were treated as negative outcomes, as were the two who relapsed. Thus, the intent-to-treat analysis produced 5 out of 19 (26%) PTSD subjects with negative outcomes, as compared to 2 out of 16 (13%) CAPS completers with negative outcomes.

The single participant who chose not to continue to the crossover arm to receive MDMA-assisted treatment sessions had a strong, sustained response to psychotherapy plus placebo (2-month CAPS = 14; LTFU CAPS = 16 and 2-month IES-R = 5; LTFU IES-R = 15) (Table 2).

**Table 2.** Global CAPS scores of individual PTSD study subjects.

Study Group	Crossover	Pre-MDMA Treatment	After two MDMA sessions (“2-month” score)	After three MDMA sessions****	LTFU	Number of months between the final MDMA treatment and LTFU CAPS
**MDMA**		43*	16		14	59.8
**MDMA**		43**	46	28	19	35.7
**MDMA**		57	0		3	65.7
**MDMA**		65	29		33	51.4
**MDMA**		76	32		18	74.3
**MDMA**		78	2	0	0	18.1
**MDMA**		89	79			NA
**MDMA**		90	16	19	60	43.0
**MDMA**		94	14		20	55.2
**MDMA**		102	60		7	46.3
**MDMA**		103	6		91	66.0
**MDMA**		113	4	2	18	17.3
Placebo	Yes	64***	15		10	40.7
Placebo	Yes	65***	27	34		NA
Placebo	Yes	70***	41	29	31	19.8
Placebo	Yes	78***	42	19	24	27.7
Placebo	Yes	84***	42	21		NA
Placebo	Yes	85***	21		17	57.6
Placebo	Yes	86***	49		14	50.4
		***Post- Psychotherapy only, Pre-MDMA		**** 3^rd^ session occurred following “2-month” outcome scores		

“NA”: not applicable, as the CAPS were not completed at LTFU by these three subjects.

* CAPS at enrollment was 56, prior to medication washout. In order to provide support during the washout period, subjects received non-drug preparatory psychotherapy, which may have led to transient decreases in PTSD symptom severity. To be conservative, the authors chose to report the post-washout CAPS scores in the initial paper and the same convention was used in this paper. ^**^CAPS at enrollment was 62, prior to the medication washout. CAPS: clinician-assisted PTSD scale; LTFU: long-term followup; MDMA: 3,4-methylenedioxymethamphetamine; PTSD: post-traumatic stress disorder.

### LTFU questionnaire results for 19 PTSD subjects

#### Degree of benefit

All subjects reported a benefit from participation in the study (median = 5, range = 3), with at least some benefit persisting (median = 5, range = 3). All participants answered “no” to the question, “Do you feel you were harmed by participation in the study?” We show the nature of the benefits reported in Table 3.

**Table 3.** Benefits endorsed from study participation, as listed in the long-term followup (LTFU) questionnaire. The 19 participants could endorse as many items as they wished.

Benefit	*n*	Percentage (%) of subjects endorsing
General well-being	17	89
Increased self-awareness and understanding	17	89
Less excessive vigilance	15	79
Less avoidance of people or places	15	79
Fewer nightmares, flashbacks or intrusive memories	13	68
Increased ability to feel emotions	13	68
Reduced anxiety	12	63
Improved sleep	12	63
Improved relationships in general	11	58
Improved relationships with spouse, partner or other family members	10	53
Enhanced spiritual life	10	53
More involved in the community/world around me	10	53
Improved mood	9	47
Increased empathy for others	8	42
Improved work performance	6	32
Increased creativity	5	26
Improved other psychological symptoms	4	21

Degree of overall benefit (Mean=4.3, Median=5)	*n*	% subjects

1 (Slight)	0	0
2	1	5
3	3	16
4	4	21
5 (Large)	11	58

Degree the benefits lasted (Mean=3.8, Median=4)	*n*	% subjects

1 Only a small amount of the benefit lasted	0	0
2 Some, but not most of the benefit has lasted	4	21
3 Most, but not all of the benefits lasted	3	16
4 Virtually all of the benefits have lasted	4	21
5 Benefits lasted and have continued to grow	8	42
		

The 16 LTFU CAPS completers reported a degree of benefit (median = 5 (maximum score possible); range = 3), similar to the degree of benefit reported by the three LTFU non-completers (median = 4; range = 3; independent Mann-Whitney *U* test, *p* = 0.58). Additionally, the 16 LTFU CAPS completers reported the same degree of persistence of benefit (median = 5; range = 3) as the three non-completers (median = 5, range = 3; independent Mann-Whitney *U* test, *p* = 0.18). These findings suggest that there were no significant differences between CAPS completers and non-completers in terms of the degree of and persistence of their benefits from the MDMA-assisted psychotherapy, reported at LTFU.

#### Participation in psychotherapy

Study subjects had been treated with psychotherapy as a pre-condition to study participation. At the time of enrollment, 16 of 19 (84%) subjects were in active psychotherapy. At LTFU, only 8 of 19 (42%) were in psychotherapy. Two continued with the same psychotherapy they had been in at the start of the study, while five were receiving a different type of psychotherapy or psychotherapy from a different therapist. Only one participant not in psychotherapy just prior to the study was attending psychotherapy at LTFU. Cognitive behavioral psychotherapy (*n* = 3) and Eye Movement Desensitization and Reprocessing (EMDR) (*n* = 4) were the most common types of psychotherapy reported at LTFU. Other psychotherapies included psychodynamic (*n* = 1) and Holotropic Breathwork (Brewerton et al., 2011) (*n* = 2).

#### Psychiatric medications

Table 4 shows the use of psychiatric medications at original study entry and LTFU. The percentage of subjects taking psychiatric medication did not change from the baseline 58% (12/19). The mean number of medicines taken fell from 1.7 to 1.3.

**Table 4.** Number of psychiatric medications at PTSD study entry (E) and LTFU (F).

Subject	SNRI/SSRI		Other anti-depressant		Anxiolytic		Sedative/ hypnotic		Anti-psychotic mood stabilizer		Other		Total		Number of months between final MDMA treatment and LTFU questionnaire
	E	F	E	F	E	F	E	F	E	F	E	F	E	F	
1*	1	1^d^			1	2^a,b^				1^c^			2	4	65.9
2		1^d^		1^d^		1^a^							0	3	73.7
3			1										1	0	55.9
4													0	0	63.97
5						1^a^							0	1	37.3
6		1^g^		1^g^									0	2	55.4
7	1	1^d^	1	1^d^									2	2	59.1
8					1								1	0	50.3
9				1^a,d^									0	1	48.9
10							1					1^f^	1	0	48.9
11	1	1^d^	1			1^b^	1			1^d^			3	3	43.7
12													0	0	9.7
13*													0	0	42.2
14	1	1^c^			1	1^a^	1	1^b^					3	3	35.2
15			3		1		1						5	0	16.0
16			1				1	2^b^			3		5	2	13.7
17	1												1	0	16.5
18	1	1^d^			2		1		1		1		6	1	16.7
19		1^e^			1		1						2	1	24.5
Total	6	8	7	4	7	6	7	3	1	2	4	1	32	23	

* relapsed to CAPS > 50 at LTFU

Reason stated on our LTFU questionnaire for taking medication: a = anxiety, b = insomnia, c = mood stabilization, d = depression, e = dealing with stressful life events, f = attention deficit hyperactivity disorder, g = not reported. CAPS: Clinician-administered PTSD scale; LTFU: long-term followup; MDMA: 3,4-methylenedioxymethamphetamine; PTSD: post-traumatic stress syndrome; SNRI: serotonin and norepinephrine reuptake inhibitor; SSRI: selective serotonin reuptake inhibitor

#### Questions about illicit drug use during LTFU period

Cannabis was the most frequently mentioned drug (*n* = 8), with reported frequency of use ranging from “once” to “occasionally” in the intervening months leading to LTFU. One participant reported having used psilocybin-containing mushrooms, but did not report the frequency. All subjects who were reporting use of these substances during the LTFU period had informed the investigators that they had also used them prior to enrollment in the original study, so these reports of use were not for new onset of use during the period leading up to LTFU. Only one participant, who had received two MDMA-assisted psychotherapy sessions, reported the subsequent use of “ecstasy” in a quasi-therapeutic setting, on a single occasion before the LTFU. Attempting a therapeutic setting similar to the study by asking a friend to be present during the session, the subject found the arrangement unsatisfactory and so reported no interest in repeating it outside a legal therapeutic context.

#### Opinion about additional sessions

All participants answered “Yes” to the question, “Do you believe more MDMA sessions would have been helpful?” for further treatment of PTSD, set either “at a later time point” or “soon after the last one.”

#### Cognitive function

In the original study, by using pre/post neuropsychological measures, we empirically demonstrated that there is no cognitive morbidity associated with the use of MDMA (Mithoefer et al., 2011). In this LTFU study, the participants subjectively reported either no change, or some improvement in their cognitive function, memory and concentration. None of the participants indicated that their “cognition (thinking), memory, or concentration” had worsened or decreased after study participation. Out of 19 participants, seven reported no change, while 13 reported improvements in these areas.

#### Participants’ comments on the LTFU Questionnaire

Of the 19 subjects who received MDMA-assisted psychotherapy, 15 wrote comments in the space provided on the questionnaire. Participants described the experimental treatment as being helpful, sometimes dramatically so (“The therapy made it possible for me to live”), but also as being difficult at times (“one of the toughest things I have ever done”). Several participants described it as a step in an “ongoing process” rather than simply a completed cure. Some comments shed light on the participants’ understanding of the way MDMA affected the therapeutic process (“It increased my ability to stay with and handle getting through emotions.” “The MDMA provided a dialogue with myself I am not often able to have, and there is the long-term effect of an increased sense of well-being.” “I was always too frightened to look below the sadness. The MDMA and the support allowed me to pull off the controls, and I … knew how and what and how fast or slow I needed to see my pain”). The full text of all comments is provided online, as supplementary material.

#### Questionnaire responses of the one subject who had not received MDMA

The subject who had a good CAPS response to psychotherapy with placebo and did not choose to receive MDMA-assisted psychotherapy reported a 4 out of 5 benefit level and a 4 out of 5 persistence of benefit on the LTFU Questionnaire. This subject, in psychotherapy at both study entry and at LTFU, was on one psychiatric medication at entry and on three psychiatric medications at LTFU.

## Discussion

The evidence we report in this LTFU study, conducted on the average of nearly 3 ½ years after the prior study’s exit date, indicates that there was an enduring, clinically meaningful benefit from MDMA-assisted psychotherapy to PTSD patients.

The fact that 3 of the 19 subjects did not complete the CAPS and IES-R must be taken into consideration in interpreting these data. These three “CAPS non-completers” did complete the LTFU Questionnaire, where they reported nearly the degree of benefit and the same degree of persistence of benefit as those who had completed the CAPS. Therefore, it may be the case that up to 89% (17/19) of those who received MDMA had long-term improvement in their PTSD symptoms. However, these three “CAPS non-completers” should be assumed to have had higher CAPS scores than the others. An intent-to-treat analysis, that made the assumption that each of these three individuals had relapsed, concluded that 74% (14/19) of these previously treatment-resistant subjects demonstrated meaningful, sustained reductions in their CAPS scores at LTFU.

At LTFU, two of the subjects who completed the CAPS had relapsed, with CAPS scores above the cutoff (≥50) that was the original study entry criterion. In other long-term follow-up investigations of PTSD treatment (listed in the introduction), relapse rates range from 0.05–41%, so this rate is comparable (from a minimum of 11% of the CAPS completers to a maximum of 26%, in the intent-to-treat analysis).

The LTFU questionnaire we developed to assess the participants, while not a validated instrument, did shed light on several important points: the apparent lack of risk of substance abuse and of neurocognitive decline, coupled with symptom improvement and other perceived benefits.

### Risk of substance abuse

The data we obtained about illicit drug use from the LTFU Questionnaire supports the hypothesis that MDMA can be administered in a clinical setting with minimal risk that the subjects will subsequently seek out and self-administer “street ecstasy,” or become dependent on the drug. This is consistent with the comments from many study subjects, who expressed the strong opinion that the therapeutic setting and close follow-up were essential elements of the treatment, and they did not think MDMA should be used without this level of clinical monitoring and therapeutic support. Furthermore, no subject developed a substance abuse problem with any illicit drug after their MDMA-assisted psychotherapy. PTSD is associated with a high incidence of co-morbid substance abuse (Brown and Wolfe, 1994; Ford et al., 2007) and MDMA, as ecstasy (which is a material that may contain other substances in addition to or instead of MDMA), has a history of being a drug of abuse, so our findings were reassuring, though not unexpected: As it is the emotional distress of PTSD that often contributes to the use of intoxicants as an escape or an attempt at self-medication (Brady et al., 2000b), when that emotional distress is reduced, as it was with this experimental treatment, it follows that the subject’s motivation for drug abuse would be reduced, as well.

### Risk of neurocognitive decline

Favorable reports about cognitive function, memory, and concentration on the LTFU Questionnaire were consistent with findings from formal measures of cognitive function that were taken before and after psychotherapy with MDMA versus placebo, in the original study. Though reports from actual testing are more reliable than reports of perceived cognitive function, this long-term self-reported evidence is still important, because of the controversy surrounding theories that there are potential risks of neurocognitive decline resulting from MDMA administration, as has been suggested by animal studies and some retrospective studies in recreational drug users (Thomasius et al., 2006; Kalechstein et al., 2007; Laws and Kokkalis, 2007; Zakzanis et al., 2007; De Sola Llopis et al., 2008; Jager et al., 2008; Schilt et al., 2008; Brown et al., 2010; Kish et al., 2010; Verbaten, 2010) and one prospective study (Schilt et al., 2007). The possible causes and precise nature of impaired cognitive function in regular ecstasy users do appear to be complex and studies have yielded contradictory findings (Gouzoulis-Mayfrank and Daumann, 2006; Rogers et al., 2009). The lack of evidence of neurocognitive decline associated with MDMA in our initial study, as well as in the LTFU self-reports are consistent with the most well-controlled studies of recreational ecstasy use and neurocognitive performance, which report largely negative findings (Halpern et al., 2004; Hoshi et al., 2007; Roiser et al., 2007; Bedi and Redman, 2008; Hanson and Luciana, 2010; Halpern et al., 2011).

### Symptom improvement and other benefits reported on our LTFU Questionnaire

It is notable that no subjects reported any harm from study participation and all of them reported some degree of benefit. Consistent with the investigators‘ clinical observations in the original study, the responses we obtained on the questionnaire indicated that participants often experienced benefits beyond decreased PTSD symptoms. This is not a radical idea; many forms of psychotherapy produce benefits in terms of psychological growth and development that are not limited to improvements in a specific disorder that may have been the original target of therapy (Tedeschi and Calhoun, 2006; Zoellner and Maercker, 2006). Such benefits may prove to be a particularly prominent and valuable feature of MDMA-assisted psychotherapy. Some of the areas of benefit that were endorsed on the LTFU Questionnaire, such as an increased self-awareness, improved relationships, an enhanced spiritual life, and more involvement in the community or world, represent effects that are not fully measured by the PTSD symptom scales.

A sustained improvement in PTSD symptoms documented on the CAPS, plus other benefits reported on the LTFU Questionnaire, have implications regarding one of the limitations of the previous study: the fact that the blinding turned out to be transparent to the investigators and to most of the participants (though not to the independent rater). This problem is inherent in all drug studies, but is particularly challenging to overcome in studies of MDMA-assisted psychotherapy, because of the prominent psychoactive effects MDMA has, as well as its effects on blood pressure and pulse rate. Observation of these effects may have contributed to the investigators’ ability to correctly distinguish MDMA-assisted sessions from therapy-only sessions (Bjorkedal and Flaten, 2011). In addition, any studies of drug-assisted psychotherapy (rather than of drug effect alone) present particular challenges in distinguishing the drug effects from the effects of psychotherapy with placebo. Nevertheless, if the placebo response is tied to receiving an apparent therapy, the favorable LTFU results we obtained a year or more later in this cohort of previously treatment-resistant subjects would suggest that the strength and duration of symptomatic improvement that we observed is not easily attributable to a placebo response or a spontaneous remission (Price et al., 2008; Benedetti and Amanzio, 2011), though further studies will be needed to establish this conclusively.

Additional evidence of the positive MDMA treatment effects is apparent in the types of benefits endorsed by the subjects on the LTFU Questionnaire and its Comments section. While not a substitute for the validated primary outcome measures, this descriptive material provides us with an important context in which the outcome data can be evaluated and understood. The benefits endorsed and described extend beyond the realm of symptom reduction. Many subjects reported deeply meaningful therapeutic experiences and ensuing improvements in their lives. A majority of participants endorsed benefits such as, “increased self-awareness and understanding” and “enhanced spiritual life.” These responses and many of the individual comments on the Questionnaire point to a subjectively authentic process of psychological and spiritual exploration and growth that could logically be expected to facilitate trauma processing and symptom reduction, and to promote healthy psychological development. Improvements of this kind could reasonably be expected to persist for a year or more, as was endorsed by 80% of the subjects, and even to “last and continue to grow,” as endorsed by 40% of subjects. In addition, several participants wrote comments describing an incomplete but ongoing process of improvement, referring to an “ongoing journey” or having “gained tools” while “still struggling in many areas.” Since these comments were usually accompanied by persistently low CAPS scores, we believe that they represent additional evidence for a meaningful, ongoing, therapeutic process that was originally catalyzed by MDMA-assisted psychotherapy and is likely leading to important benefits in addition to PTSD symptom reduction. On the other hand, these comments could be taken to indicate a weakness in the treatment, as evidenced by continued psychological challenges; however, they more likely convey a strength of the treatment, in that it can enhance participants‘ capacity to engage more effectively in their own continued healing and growth.

### Limitations

There are several limitations of the study design to be taken into account in interpreting our data. Only 16 out of 19 subjects completed the LTFU CAPS or IES-R, though this issue was addressed with the intent-to-treat analysis and with the information gathered from all 19 MDMA subjects who did complete the Questionnaire. While the randomized control group design, along with the crossover arm in the original trial, went a long way toward ruling out the possibility that there was only a placebo response, there was no meaningful control group for our LTFU, because all but one subject ultimately received active treatment in the initial study. Because it is not practical nor ethical to maintain a placebo group for over a year, nor to control for other treatments for that period of time, this limitation is commonly found in LTFU studies. In light of this limitation, it is possible that the favorable results of this LTFU study were caused by resolution of symptoms due to natural history (Price et al., 2008) or to other variables that were not controlled for after the original 2-month follow-up. An important argument against this is that the cohort was originally screened specifically to have *treatment-refractory* PTSD and their mean duration of symptoms had been more than 19 years.

The long follow-up period allowed us to identify relapses that would not have been identified within a shorter follow-up period. On the other hand, the longer the time elapsed between the experimental intervention and follow-up, the more likely it is that life events will have intervened and had their own effect on outcome, either positive or negative. The interval between final MDMA-assisted session and LTFU varied considerably because the LTFU was an amendment to the protocol written and approved well after the start of the study, with the first LTFU assessments occurring 5 years after study initiation. While this enabled us to obtain follow-up data at exceptionally long time intervals after treatment, it would have been preferable to have measured long-term data at a fixed interval from each subject’s final MDMA session. Ongoing studies of MDMA-assisted psychotherapy now include a long-term follow-up component from the start that will result in far less variation in time of LTFU. In addition, we believe that future versions of the LTFU questionnaire should include questions about significant intervening life events.

Perhaps the most important factor confounding the attribution of the persistence of therapeutic benefits entirely to our experimental intervention is that at LTFU, 8 of 19 subjects were still in psychotherapy and 12 of 19 were taking psychiatric medicines. Although this did represent a decrease in the use of psychotherapy and psychiatric drugs compared to baseline, it is impossible to know how much these treatments may have influenced the durability of benefits measured at LTFU. The small sample size does not allow for meaningful statistical comparisons between the subjects who continued treatment and those who did not. Although these confounding factors preclude a definite conclusion about the long-term benefits of the experimental treatment, it is noteworthy that the original sample included only participants with PTSD, usually of many years duration, who had already proven resistant to prior therapy and medication. We believe it unlikely that additional medication and/or psychotherapy similar to what they had received prior to entry in our first study would have consistently led to any sustained improvement across the majority of participants, if there had not been some significant lasting therapeutic effect and benefit from our MDMA-assisted psychotherapy sessions; however, it may well be that, for some participants, ongoing medication and/or psychotherapy were important factors in maintaining remission, analogous to the way antidepressants may be necessary to maintain remission following electroconvulsive therapy (ECT) in patients who had previously been treatment resistant (Sackeim et al., 2001; Sienaert, 2011).

There are several other possible explanations for the substantial rates of continued psychotherapy and psychiatric medications at LTFU in those subjects with persistently low CAPS scores: the CAPS could have failed to capture some PTSD symptoms, though presently there is no better instrument to do so; ongoing treatment could have been targeted at a psychopathology other than PTSD, such as other anxiety disorders or persistent or recurrent mood disorders; and medications could have still been prescribed in the absence of an active Axis I disorder or other well-established indication. Alternatively, with the amelioration of their PTSD psychopathology, it may be that participants were undergoing psychotherapy to further their personal growth and self-understanding and/or to support expansion and integration of any therapeutic gains made during MDMA-assisted psychotherapy. The reasons given by subjects for the use of psychiatric medications lend some support to the notion that most drugs were not prescribed for PTSD, but the Questionnaire did not ask for the reasons for ongoing psychotherapy.

Attempts are under way to further address these limitations and to build upon the results. The authors are currently conducting a study of military veterans with PTSD, using a randomized, three-arm design with low, medium, and full dose MDMA, in order to address the limited effectiveness of the blind in the original study. Steps are also being taken to capture more information about other possible beneficial effects by inclusion of the Posttraumatic Growth Inventory (Tedeschi and Calhoun, 1996) and a measure for mystical or transformative experiences (Griffiths et al., 2006), as well as through collaboration with others who are applying process measures and descriptive analysis to video recordings from these studies.

The investigators have recently obtained approval for a new proof-of-principle study to determine if one additional open-label MDMA-assisted psychotherapy session can restore prior therapeutic gains in people who had responded well to MDMA-assisted psychotherapy initially, but had relapsed a year or more later. Studies are also being undertaken by MAPS-sponsored researchers at other locations, to determine whether or not these results can be replicated. Valuable data could be generated if additional studies were conducted by independent research teams using the same treatment method described in the Manual; plus in the future, other treatment methods using MDMA should also be tested. Future studies will be needed to finalize the double-blind methodology, assess the magnitude and variance of the treatment’s effect if delivered by other co-therapist teams, evaluate whether the method is generalizable across patient populations and different cultures, and address questions about the therapeutic method’s mechanism of action, optimal dosing and possible refinements to the method.

## Conclusions

Over a decade ago, we conceived this study in an attempt to develop and test a novel therapy for PTSD, a clinical condition for which a wider array of effective treatment options has remained a crucial need. Using rigorous scientific methodology, we describe a potentially beneficial new therapeutic modality with support from solid empirical data. Following this promising first step, we now report very long-term outcome results in the original cohort, demonstrating a sustained benefit over time, with no cases of subsequent drug abuse and no reports of neurocognitive decline. These results indicate that there was a favorable long-term risk/benefit ratio for PTSD treatment with just a few doses of pure MDMA administered in a supportive setting, in conjunction with psychotherapy. Should further research validate our initial findings, we predict that MDMA-assisted psychotherapy will become an important treatment option for this very challenging clinical and public health problem.
